# Robot Programming from a Single Demonstration for High Precision Industrial Insertion

**DOI:** 10.3390/s23052514

**Published:** 2023-02-24

**Authors:** Kaimeng Wang, Yongxiang Fan, Ichiro Sakuma

**Affiliations:** 1 FANUC Advanced Research Laboratory, FANUC America Corporation, Union City, CA 94587, USA; 2 Department of Precision Engineering, The University of Tokyo, Tokyo 113-8654, Japan

**Keywords:** programming by demonstration, robotic insertion, visual servoing, line matching

## Abstract

We propose a novel approach for robotic industrial insertion tasks using the Programming by Demonstration technique. Our method allows robots to learn a high-precision task by observing human demonstration once, without requiring any prior knowledge of the object. We introduce an Imitated-to-Finetuned approach that generates imitated approach trajectories by cloning the human hand’s movements and then fine-tunes the goal position with a visual servoing approach. To identify features on the object used in visual servoing, we model object tracking as the moving object detection problem, separating each demonstration video frame into the moving foreground that includes the object and demonstrator’s hand and the static background. Then a hand keypoints estimation function is used to remove the redundant features on the hand. The experiment shows that the proposed method can make robots learn precision industrial insertion tasks from a single human demonstration.

## 1. Introduction

With the shifting of manufacturing from large-scale production to small-batch customized production, the traditional robot programming method is difficult to meet the market needs due to the requirements of highly-skilled robotic experts and time-consuming procedures. Therefore, developing an intuitive and efficient robot programming method that can quickly deploy robots in a novel environment becomes much more important [1].

Programming by Demonstration (PbD) attracts a lot of attention in this context as it allows users to program new tasks on a robot through a demonstration without requiring expertise in robotics [2]. Compared to kinesthetic or teleoperation teaching, which may prevent free movement of the demonstrator’s hand, vision-based imitation learning is a more natural approach for PbD since users can demonstrate the desired behavior without the extra interactive training to adapt to robot operation. Despite these considerable advantages, the commercial high-precision application of vision-based imitation learning in robotic assembly is still not mature. Some methods, such as behavioral cloning methods, observe human movement from visual input and learn a mapping between perception results and actions. A 6D object pose tracker relying on neural network training [3] and an object tracker with manually designed visual features [4] have been introduced to achieve high-precision assembly tasks. However, it is highly challenging to train a robust and accurate object pose tracker and quite time-consuming to collect training data or to manually specify visual features for each task. Other methods, such as reinforcement learning, may be used to eliminate the uncertainty of positioning errors by self-supervised exploration of the environments. However, it requires significant effort from a human operator to manually reset the environment while the failures occur during real-world training. Therefore, there are several challenges in applying vision-based imitation learning in the commercial application of robotic assembly: 1. the robust and accurate object perception system is required for high-precision positioning; 2. the amount of human-supervised interaction is necessary to program a new task in different environments.

**Figure 1:**
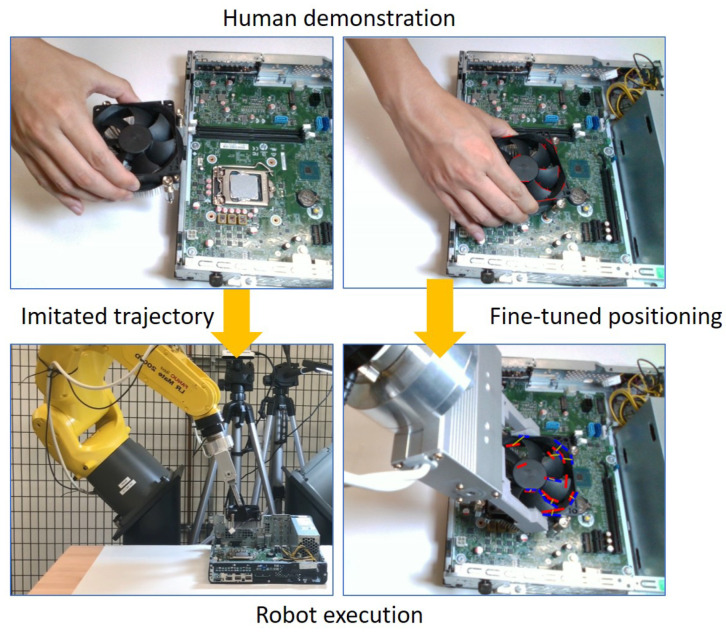
Overview of the proposed Programming by Demonstration approach. An imitated trajectory is generated by tracking human hand movements, as well as the visual features are collected and used for high-precision positioning through the use of visual servoing.

In this work, we introduce a novel approach for high-precision robotic industrial insertion tasks by combining Programming by Demonstration and visual servoing, as shown in Figure 1. The imitated trajectory is directly generated according to human hand movements, and the accurate target position is fine-tuned by the visual servoing technique. In contrast to the pure visual servoing approach, the proposed integration method may avoid sub-optimal or unfeasible trajectories that failed in a collision by learning the trajectory directly from human demonstration. In general, visual servoing requires users to manually design image features (keypoints, lines, circles, etc.) which are not easy for novice users. For automatically identifying the image features on the object, we localize object position through the use of the moving object detection approach [5] without requiring any hand engineering. In addition, a new line feature matching method is proposed to solve the lighting or background-changing problems that are often encountered by traditional feature descriptors (SIFT [6], etc.). In summary, our main contributions are:

- We propose a novel Programming by Demonstration (PbD) framework to achieve a high-precision robotic industrial insertion task by combining visual servoing techniques, which generate imitated trajectory from human hand movements and then fine-tune the target positioning using the visual servoing.
- We simplify the object localization problem as a moving object detection problem. This allows our method to automatically identify the image features on the object without requiring tedious handcrafted image feature selection and any prior knowledge of the object.
- We introduce a new line feature matching approach, instead of traditional feature descriptors that are often affected by lighting or background changing, for identifying association constraints between demonstrator image and imitator image.

## 2. Related Works

**Behavioral cloning methods.** This type of approach provides users an intuitive programming way to make robots reproduce the desired movements that humans did by perceiving human behaviors from visual input [7]. Gaussian mixture models (GMMs) [8], Hidden Markov models (HMMs) [9], or Dynamic movement primitives (DMPs) [10] are popular methodologies to encode the human movements as parameterized action primitives. To achieve high-precision positioning in assembly tasks, such as installing a component into a slot, estimating the 6D pose of the workpieces is also required. The traditional methods localize object positions with 3D CAD model matching, which often fails when objects are occluded by hands or other objects in assembly cases. By leveraging recent advances in deep learning, a number of efforts learn a more robust object pose estimator using a large dataset [11]. However, it is cumbersome to prepare the training data for each object. In contrast, the proposed method models object localization as a moving object detection problem to extract image features used for visual servoing without requiring any prior knowledge of the object.

**Reinforcement learning methods.** This type of approach makes it possible to achieve sophisticated goals in uncertain environments by training a policy to maximize a specified reward function [12]. However, reinforcement learning typically learns a new task from scratch, which requires a large number of trials. Interactive Reinforcement Learning addresses the sample inefficiency problem by allowing an agent to improve its behavior from interaction with a human trainer [13], which is successfully applied in the metal toy locomotive assembly [14]. Inverse reinforcement learning, which tries to extract a reward function from the demonstrations, is proposed to solve the difficulty in manually designing a reward function [15]. In [16], the method to learn time-invariant reward functions via inverse reinforcement learning is proposed to accomplish the peg-hole insertion task. While combining imitation learning with reinforcement learning [17] has yielded great success in many tasks, it requires either a mount of human demonstrations [18] or tedious human-supervised interaction to manually reset failures during the real-world training [19]. In contrast, our method requires only watching the demonstration once without a specific manual environment setup.

**Feature-based methods.** This type of approach provides an explainable and stable representation of perception of the demonstrations using image features [20]. Such approaches try to balance intuitive programming and precise positioning with the combination of imitation learning and visual servoing. A key difficulty in using visual servoing is image feature extraction from human demonstrations without handcrafted feature assignments. To overcome this problem, recent works encode image observations from the human demonstration in image features by leveraging advanced deep neural network [21,22]. However, a large number of human demonstration data is required for training, and the end-to-end approach lacks explainability for users to tune when a failure occurs. In contrast, our method identifies the image features on the object using the moving object detection technique without requiring any prior knowledge of the object.

**Line feature matching.** Visual servoing is highly dependent on a robust image feature matching method to find correspondences between a target image from the human demonstration phase and a current image from the robot execution phase. However, the traditional appearance-based methods (e.g., LBD [23]) are less robust to illumination and viewpoint changing since the corresponding lines are obtained based on the texture around each line segment. Iterative Closest Point (ICP) is a well-known technique to solve the rigid alignment between two point clouds. A method named Iterative Closest Line (ICL) [24], inspired by the ICP algorithm, was proposed to estimate rotation and translation between two line segment sets in 3D space. [25] further improves the line-matching accuracy by using the Random Sample Consensus (RANSAC) approach. However, the alignment accuracy is sensitive to depth noise that comes from sensor measurement. To overcome this limit, we propose a novel line feature matching approach using 2D and 3D information. The line feature matching problem is formulated as an optimization problem, which minimizes a cost function with respect to direction and distance between corresponding line segments in 2D image space.

## 3. Proposed Approach

**Figure 2:**
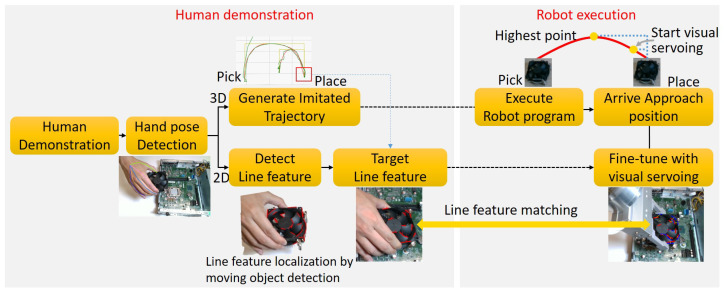
The pipeline of the proposed approach.

The proposed system consists of two phases: the human demonstration phase and the robot execution phase, shown in Figure 2.

In the human demonstration phase, the demonstrator’s hand movements are collected with a 3D camera or from a serial RGBD video. We generate imitated trajectories in Cartesian space by cloning hand movements. At the same time, the object position in the 2D image is localized through the use of the moving object detection technique and then line features on the object are recorded as the target line features, which are used for visual servoing.

In the robot execution phase, first, the robot moves to the goal position by following the imitated trajectories generated from the demonstrator’s hand movements. When the robot moves close to the goal position, the visual servoing control is triggered for precision placement. The line feature correspondences between the target image from the human demonstration and a current image of the robot execution are estimated using a novel line feature matching approach described in Section 3.2.2.

### 3.1. Human Demonstration Phase

#### 3.1.1. Imitated Trajectory Generation

**Figure 3:**
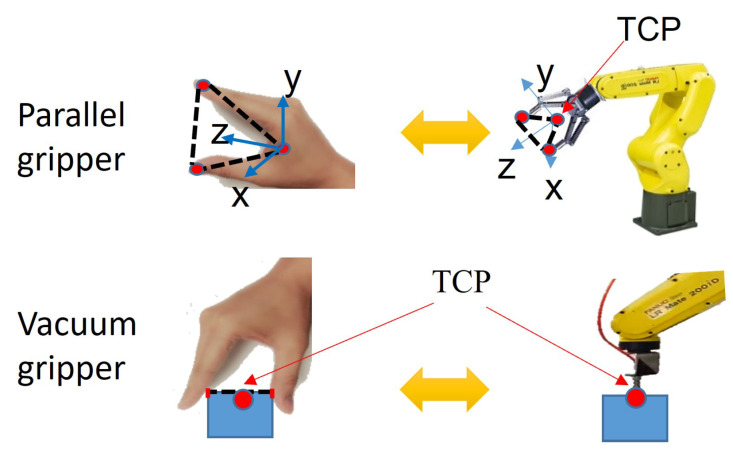
The hand gesture mimics both parallel and vacuum grippers.

The hand gesture formed by the thumb and index fingers is used to mimic either parallel gripper or vacuum gripper, shown in Figure 3. From this hand gesture, both the robot grasping pose and the robot trajectories in Cartesian space can be generated by simply copying the demonstrator’s hand movements. To track the demonstrator’s hand movements, we train a convolutional neural network with the ResNet101 structure to infer 2D keypoints of the hand similar to [26] and then retrieve the depth value corresponding to the 2D keypoint pixels to calculate 3D coordinates of each hand keypoint.

After collecting all data on hand movements from observation of the demonstration, Dynamic Movement Primitives (DMPs) [27] are used to encode the hand movements as a spring damping system offline and generate smooth robot trajectories online. Note that the generated imitated trajectory only leads the robot to move close to the goal position. The threshold $d_{vf}$, the distance from the robot to the goal position, is defined to indicate the timing when the robot controller switches to the visual servoing mode.

#### 3.1.2. Line Feature Generation

Visual servoing controls the robot moving to the target position through the use of the corresponding image features between the target image and the current image. Keypoints and line segments are the most basic image features used in visual servoing. Due to the low-textured characteristics in industrial environments, the keypoints may be difficult to be detected in such scenes. In contrast, line features usually exist on the edges of the object or the intersection of planes which can be detected even in low-textured environments. Furthermore, the precision of matching between lines is higher than keypoint matching due to their distinctiveness. Therefore, we select line features as image features for visual servoing in our work. Line segments on the workpiece surface in the target image and the current image can be extracted by the Line Segment Detector approach [28].

Instead of manually specifying image features, our method can automatically identify the features on the surfaces of the object for visual servoing. The positions of the object are localized in the image space using the moving object detection approach without requiring CAD models or additional training processes. Detection of the moving object is useful in various applications, like visual surveillance or background substitution. The Background subtraction (BGS) approach [5] is used by moving object detection for modeling static objects as a background and dynamic objects as a foreground in a scene. The color-based BGS technique has good accuracy, but it is sensitive to illumination changes. On the other hand, the depth-based BGS technique is not affected by illumination, but it often experiences sensing noise problems. To handle the problems, we merge complementary information of both RGB and depth information to obtain better segmentation results.

**Figure 4:**
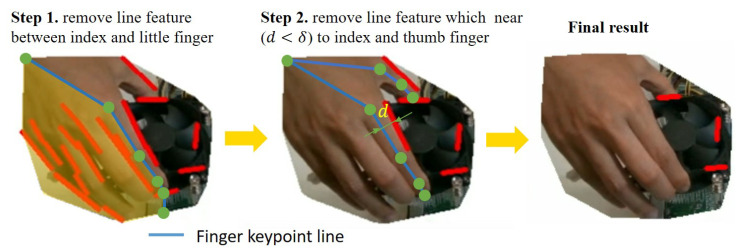
Remove noise line features that belong to the hand.

**Figure 5:**
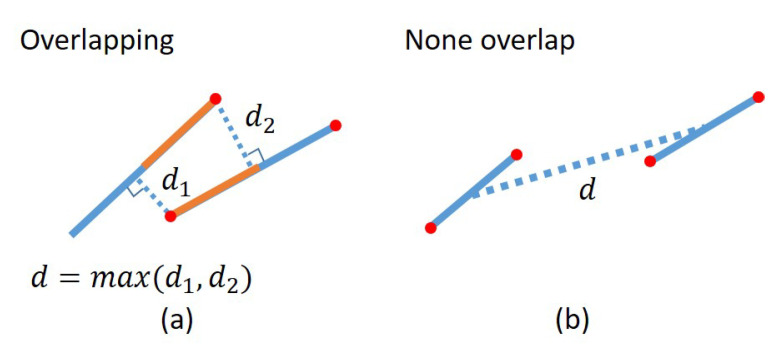
Line distance measurement between two line segments in a 2D image. (**a**) Overlapping case: Calculate the perpendicular distance between each overlapping endpoint and line segment, and take the maximum $d=max(d_{1},d_{2})$ as the distance. (**b**) Non-overlapping case: Calculate the distance between the center point of each line segment.

Since the demonstrator’s hand also moves together with the object during the demonstration, both the hand and the object are considered moving objects to be segmented by the moving object detection approach. Therefore, we further remove line features on the hand using the hand keypoints detection function described in Section 3.1.1. Figure 4 shows the process of how identifying the line features on the hand. First, all the line features between the index finger and the little finger are removed. Then, the distances between each line feature and finger keypoint line that the line segment between two hand joints are calculated. The distance between two line segments is defined in Figure 5. We use two constraints in the form of overlap and non-overlap to measure the distance similarity between lines. If the two lines overlap in 2D image space, which may intersect another line segment along a perpendicular vector, the maximum distance $d=max(d_{1},d_{2})$ at each overlapping endpoint is measured. If the two correspondence lines do not overlap in 2D image space, the distance from the center of the two line segments is measured. If the measured distance is smaller than the threshold $d_{h}$, we consider it belongs to hand and remove it. Finally, only the line features belonging to the object are collected and stored for visual servoing control in the robot execution phase.

### 3.2. Robot Execution Phase

#### 3.2.1. Fine-Tuned Trajectory Generation

When the robot moves to the position that is $d_{vf}$ away from the goal position according to hand movements, the visual servoing control is triggered to be executed to fine-tune the final placement position.

#### 3.2.2. Line Feature Matching

**Figure 6:**
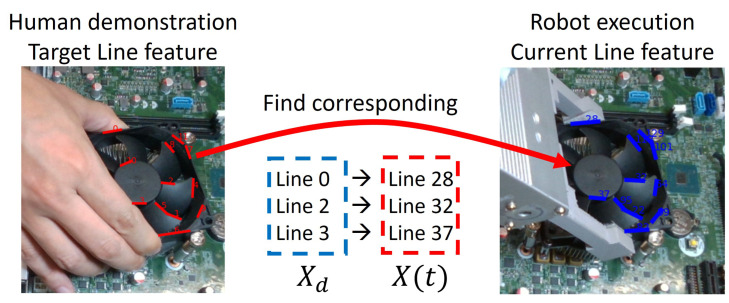
Line matching between the target image and current image for visual servoing.

The proposed line feature matching approach consists of three steps to find the correspondence line segments between the target image and the current image. The target image is taken in the human demonstration when the workpiece reaches the goal position, and the current image is taken in each robot control cycle which is being performed using visual servoing, as shown in Figure 6. First, generate the initial line pairs by measuring the similarities according to the direction and distance between each correspondence line segment. Second, randomly select four pairs from the initial line pairs, and calculate the optimal transformation matrix *T* by solving an optimization problem. Third, validate a total error of all paired lines based on the obtained transformation matrix *T* which was calculated in the previous step. If the total error is smaller than a threshold δ, the transformation matrix *T* is taken as the final result. Otherwise, go back to the second step and loop again.

The first step is to generate initial line pairs by measuring similarities according to the differences in the angle and the distance between each line pair. To begin with, we measure the angle between two line segments to create an ordered line pair list using Equation (1).

(1) $$\theta =arccos(\frac{l^{tar}\cdot l^{cur}}{\left|\right|l^{tar}\left|||\right|l^{cur}\left|\right|})$$

**Figure 7:**
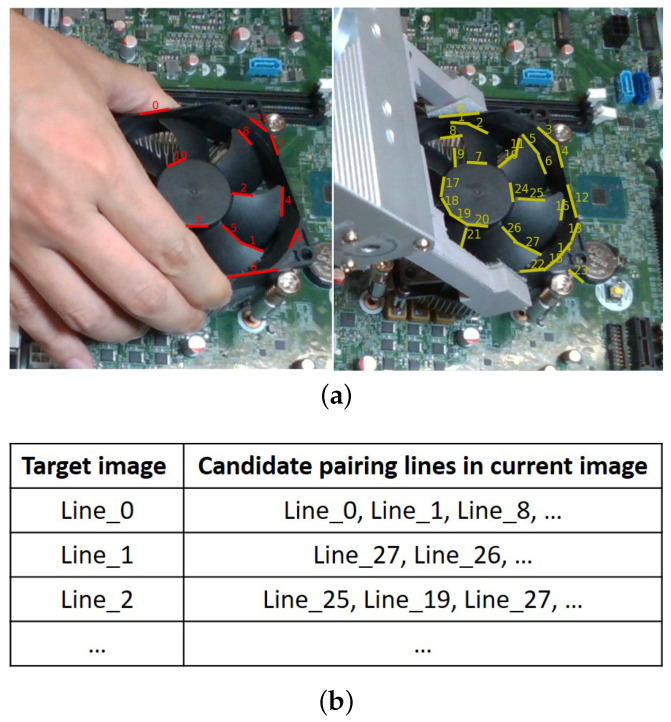
(**a**) Detected line segments in the target image (**left**) and the current image (**right**). (**b**) Initial paired line segments between target and current images. Initial line pairs based on the angle and distance difference.

For example, the angles in 2D image space between Line $l_{n}^{tar}$ in the target image and each line $\left(l_{1}^{cur},l_{2}^{cur},\cdots ,l_{m}^{cur}\right)$ in the current image are calculated. Based on angle similarity results the rank order is created that lists the line pairs in order of increasing angular difference, as shown in Figure 7. Second, the distance defined in Figure 5 is used to measure the distance similarity from each line in the target image to each line in its rank-ordered list obtained from the angle similarity. A pair is chosen as an initial candidate line pair if it has the shortest distance as well as the angle difference is smaller than a threshold $\theta_{t}$.

**Figure 8:**
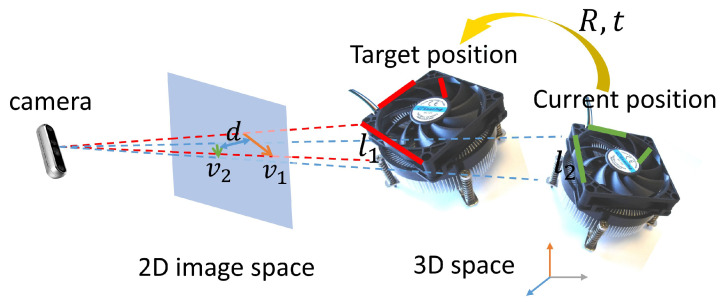
Find the optimal *R* and *t* by minimizing the direction between $v_{1}$ and $v_{2}$ and distance *d*.

The second step is to randomly select four line pairs from the initial pairs, and solve the corresponding problem as an optimization problem, which is inspired by the ICL algorithm, to find the optimal transformation. The ICL algorithm searches for the optimal transformation matrix *T* composed of a 3D rotation *R* and a 3D translation *t* to align target lines and current lines. Figure 8 shows an example that transforms the line segments in the target image to the current image with obtained *R* and *t*. To solve *T*, ICL minimizes the angle between the correspondence lines that have to be parallel and the distance of these correspondence lines. More specifically, the equations are written as

(2) $$\begin{array}{cc} R & =\underset{R}{\arg\min}\sum_{i=1}^{N}||R\cdot {}^{i}v_{3d}^{tar}-{}^{i}v_{3d}^{cur}{\left|\right|}_{2}^{2} \end{array}$$

(3) $$\begin{array}{cc} t & =\underset{t}{\arg\min}\sum_{i=1}^{2N}||R\cdot {}^{i}p_{3d}^{tar}+t-{}^{i}q_{3d}^{cur}{\left|\right|}_{2}^{2} \end{array}$$

where ${}^{i}v_{3d}^{tar}$, ${}^{i}v_{3d}^{cur}\in R^{3}$ denote the line direction vectors of a correspondence pair between target lines and current lines, and ${}^{i}p_{3d}^{tar}$, ${}^{i}q_{3d}^{cur}\in R^{3}$ denote correspondence points on the line pair which the direction vectors are ${}^{i}v_{3d}^{tar}$, ${}^{i}v_{3d}^{cur}$, respectively.

ICL algorithm, however, is sensitive to the accuracy of depth measurement, which requires a high-performance 3D camera that usually causes an additional cost. We propose a novel algorithm that eliminates the effects of noise by projecting the 3D line segments onto the 2D image plane and then minimizing the following cost function in the 2D space.

(4) $$R,t=\underset{R,t}{\arg\min}\left(E_{a}+\lambda E_{d}\right)$$

The first term $E_{a}$ evaluates the direction similarities of each line pair and is defined as

(5) $$\begin{array}{cc} E_{a} & =\sum_{i=1}^{N}{∥{}^{i}v_{2d}^{tar}-{}^{i}v_{2d}^{cur}∥}_{2}^{2} \end{array}$$

(6)whereiv2dtar=i1p2dtar−i2p2dtar(7)p2dtar=K·[Rt]·p3dtar(8)iv2dcur=i1q2dcur−i2q2dcur(9)q2dcur=K·q3dcur

where ${}^{i}v_{2d}^{cur}\in R^{2}$ is the normalized direction vector of the line in the current image, ${}^{i}v_{2d}^{tar}\in R^{2}$ is the normalized direction vector of the corresponding line in the target image after being transformed with rotation *R* and translation *t* by Equation (7). $p_{2d}^{tar},q_{2d}^{cur}$ are the endpoints of the line segments in the target image and the current image, $p_{3d}^{tar},q_{3d}^{cur}$ are 3D positions corresponding to the 2D endpoints $p_{2d}^{tar}$ and $q_{2d}^{cur}$. *K* is the camera intrinsic parameters and i∈N,N is the total number of line pairs respectively. Note the direction of all the line segments is normalized between $\left[-\frac{\pi }{2},\frac{\pi }{2}\right]$.

The second term $E_{d}$ evaluates the distance similarities of each line pair and is defined as

(10) $$\lambda E_{d}=\lambda \sum_{i=1}^{N}{∥{}^{i}d_{2d}^{tar}-{}^{i}d_{2d}^{cur}∥}_{2}^{2}$$

where λ is a weight parameter to balance the two terms $E_{a}$ and $E_{d}$. ${}^{i}d_{2d}^{tar}$ and ${}^{i}d_{2d}^{cur}$ are defined as the perpendicular distance of a line from the origin in the 2D image coordinates using the following equation.

(11)d=|c|a2+b2(12)a=y2−y1(13)b=x1−x2(14)c=x2y1−x1y2

where a,b,c are coefficients which describe a line segment as equation ax+by+c=0. a,b,c can be obtained by using the endpoints $\left(x_{1},y_{1}\right),\left(x_{2},y_{2}\right)$ of each line segment.

We use the Levenberg–Marquardt algorithm [29] to solve the cost function Equation (4). Since not all the initial line pairs are correct, similar to RANSAC if the result of $E_{a}+\lambda E_{d}$ does not converge, we repeat the process to randomly pick up another four candidate line pairs from initial line pairs until $\left(E_{a}+\lambda E_{d}\right)$ is less than the threshold δ.

**Figure 9:**
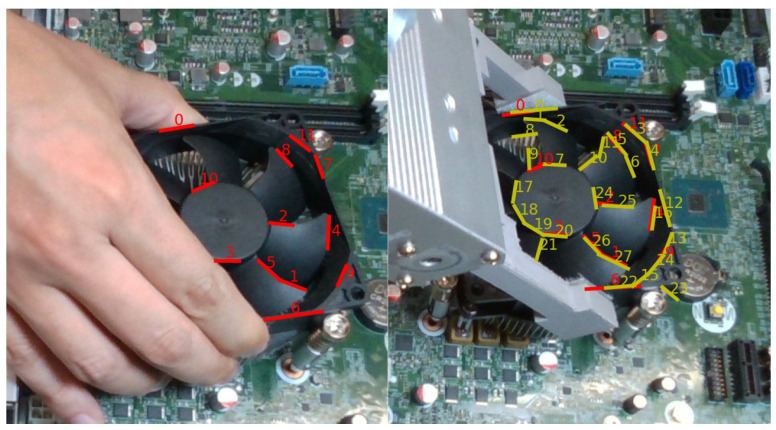
Transform the line segments in the target image (**left**) to the current image (**right**) with obtained *R* and *t*.

**Figure 10:**
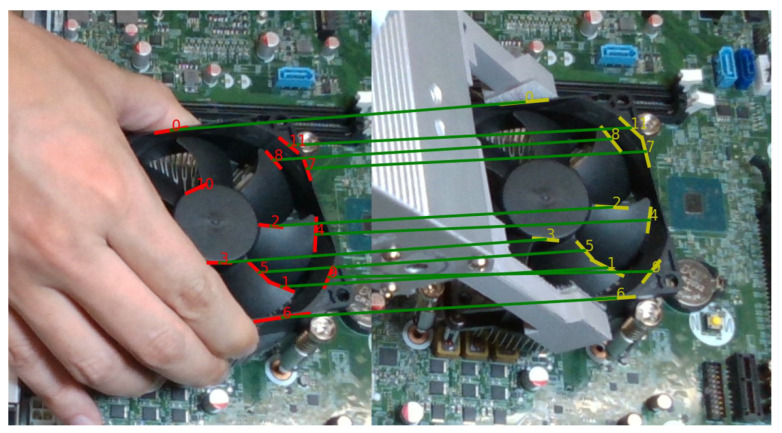
The final result of the paired line segments. The green lines indicate the paired line segments between the target image (**left**) and the current image (**right**).

The third step is to re-pair all lines based on the obtained *R* and *t*, which transform lines $l_{tar}$ in the target image to $l_{tar}^{\prime }$ via Equation (7), as shown in Figure 9. The new line pairs are determined by measuring the similarities between $l_{tar}^{\prime }$ and $l_{cur}$ using the same method introduced in the first step. We take line $l_{tar}$ and $l_{cur}$ to be a new matched line pair if their similarity $E_{a}+\lambda E_{d}$ is less than the threshold δ. However, if the total number of matched line pairs is smaller than max(4,N/2) where N is the number of line pairs, the result of Step 2 may be unreliable. Then, the process loops back to the second step to calculate new *R* and *t*. Figure 10 shows the final result of the correspondence line segment between the target image and the current image. The full algorithm of the line feature matching is summarized in Algorithm 1.
**Algorithm 1** Line Feature Matching1:**Input:**$p_{i}\in l^{tar},q_{i}\in l^{cur}$2:**Output:**Matched line segment pairs$FinalPair_{p,q}$3:**Step 1:**Initialize$InitPair_{\left(p_{i},q_{j}\right)}$4:**for**each$p_{i}\in l^{tar}$**do**5:     **for** each $q_{j}\in l^{cur}$ **do**6:        **if** $|angle\left(p_{i},q_{j}\right)|\leq \delta_{\theta }$ **then**7:            $InitPair_{p_{i}}\leftarrow InitPair_{p_{i}}\cup q_{j}$8:     $InitPair\leftarrow InitPair\cup (argmin(distance\left(p_{i},q_{j}\right),$**for**j=1,...,N))9:**while maxIterations not reached do**10:     **Step 2:** Calculate R,t11:     $\mathrm{select}\mathrm{randomly}{\left(p_{i},q_{j}\right)}_{n=1,...,N}\subset InitPair_{(}p_{i},q_{j})$12:     $R,t\leftarrow min_{R,t}E\left(R,t\right)$13:     **Step 3:** Obtain $FinalPair_{p,q}$14:     Initialize n=0, **and empty buffer** $TempPair_{p,q}$15:     **for** each ${\left(p,q\right)}_{i}\in InitPair_{(}p,q)$ **do**16:        **if** $E_{R,t}{\left(p,q\right)}_{i}\leq \delta_{E}$ **then**17:            n←n+118:            $TempPair_{p,q}\leftarrow TempPair_{p,q}\cup \left(p,q\right)$19:     **if** $n_{i}>maxInlier$ **then**20:        $maxInlier\leftarrow n_{i}$21:        $FinalPair_{p,q}\leftarrow TempPair_{p,q}$22:**end while**23:**return**$FinalPair_{p,q}$

#### 3.2.3. Visual Servoing

The visual servoing technique is employed to drive the errors between the matched line segments to zero. The control schemes [30] is typically defined by

(15) $$\begin{array}{cc} e(t) & =s_{c}\left(t\right)-s_{t} \end{array}$$

(16) $$\begin{array}{cc} v_{c} & =-\alpha L_{s}^{†}e\left(t\right) \end{array}$$

where the vector $s_{t}$ is a set of the target image features, and the vector $s_{c}\left(t\right)$ is the corresponding image features extracted from the current image at the time step *t*. The velocity of the camera $v_{c}$ is related to the feature errors e(t) by the interaction matrix $L_{s}$. The line feature is defined in the cylindrical coordinates which $\rho =\sqrt{x^{2}+y^{2}}$ and θ=arctan(y/x). Thus, the interaction matrix $L_{s}$ is given by

(17) $$L_{\left(\rho ,\theta \right)}=\left[\begin{array}{cccccc} \frac{-c}{Z} & \frac{-s}{Z} & \frac{\rho }{Z} & (1+\rho^{2})s & -(1+\rho^{2})c & 0\\ \frac{s}{\rho Z} & \frac{-c}{\rho Z} & 0 & c & s & -1 \end{array}\right]$$

where c=cosθ, s=sinθ and *Z* is the corresponding depth value. To control the 6 dimensions of $v_{c}$, at least three line segment pairs are needed which $L_{s}=\left[L_{(\rho ,\theta )1},L_{(\rho ,\theta )2},\cdots ,L_{(\rho ,\theta )n}\right]$, n≥3.

## 4. Experiment

In this section, we first evaluate the proposed line feature matching in the simulator and then conduct four high-precision tasks to evaluate the effectiveness of our PbD-based approach on a FANUC 6 Degree-of-Freedom (DoF) industrial robot.

### 4.1. Line Feature Matching

**Figure 11:**
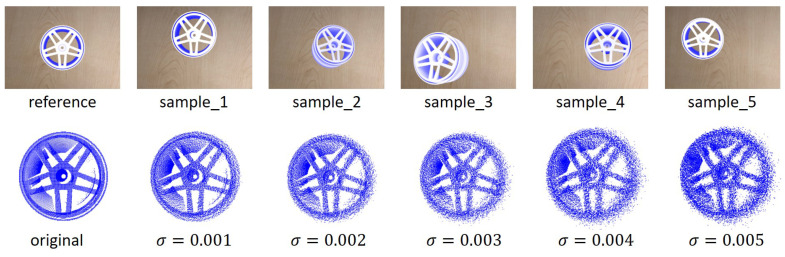
The upper row is the rendered images with 5 different camera viewpoints. The lower row is the depth data with different standard deviations σ from 0 to 0.005.

To evaluate the proposed line feature matching method, a dataset using the 3D CAD model from dataset MVTec ITODD [31] is created. 3D computer graphics software Blender [32] is used to render 2D images with different camera viewpoints. The object is transformed randomly as a rigid motion within the camera field of view, constraining the rotation as Euler angles in the range $\left[\pm 10^{\circ },\pm 10^{\circ },\pm 10^{\circ }\right]$ and the translation in the range [±50 mm, ±50 mm, ±50 mm] for obtaining 5 different patterns, as shown in Figure 11. Gaussian noise with zero means and σ standard deviation is added to the point cloud of the 3D CAD model. Five noisy level datasets that increase from σ=0 to σ=0.005 are generated for each pattern. The success rate of all found line pairs is defined as the evaluation metric used to compare to the ICL method.

**Figure 12:**
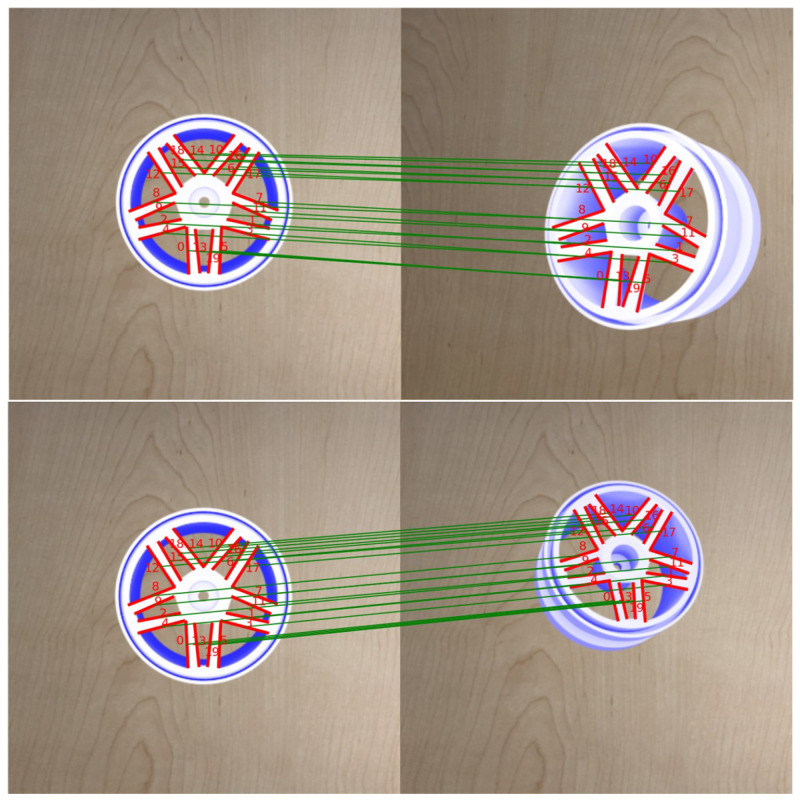
Line matching results of the proposed method. The left side shows the target image with detected line segments. The right side shows the current image. The green lines connect the correspondence line pairs between the target and current images.

**Figure 13:**
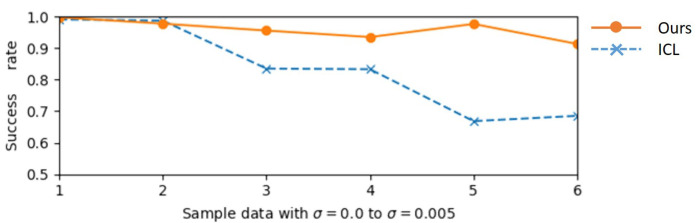
Comparison between the ICL method and our proposed method.

Figure 12 shows the results of the proposed line-matching method. The comparison result between ICL and the proposed method is shown in Figure 13. Both methods give good performance while the intensity of the Gaussian noise is small. However, the ICL method is highly affected by the noise level since the translation computation is only dependent on the accuracy of the 3D position of line segments. In contrast, the proposed method gives a higher success rate in most cases. This is thanks to the cost function that directly minimizes the errors in 2D space which reduces the depth noise impact.

### 4.2. Experiments with Real Robot

**Figure 14:**
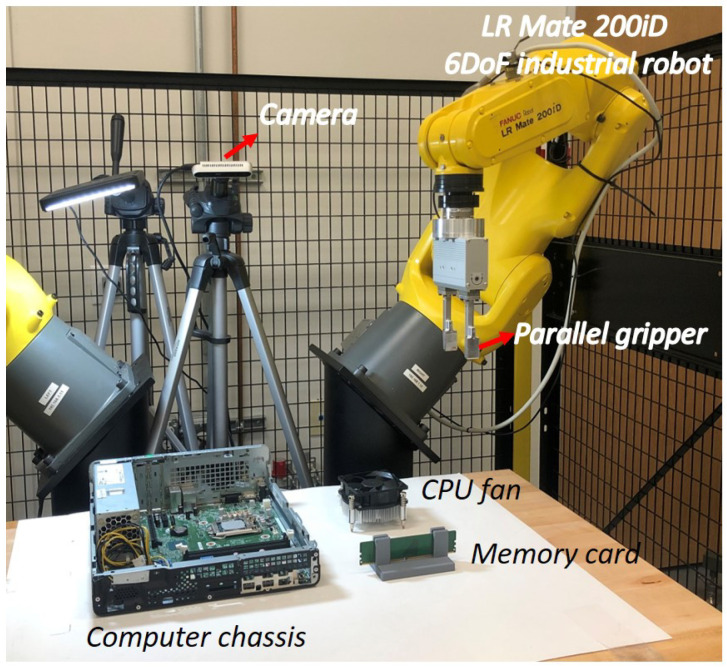
Experimental setup.

The experimental setup consists of one FANUC LR-Mate 200iD 6 DoF industrial robots and one Intel RealSense D435 camera. The robot is equipped with an SMC parallel gripper for grasping the object. The camera is fixed on the ground to configure the eye-to-hand system. Since the same workspace is shared by the human demonstrator and the robot, we use the same camera in both the human demonstration phase and the robot execution phase. Figure 14 shows the experimental setup. The calibration of the camera setup is pre-defined that consists of the intrinsic parameters of the camera, the transformation matrix between the base frame of the robot and the camera, and the transformation matrix between the table and the camera.

**Figure 15:**
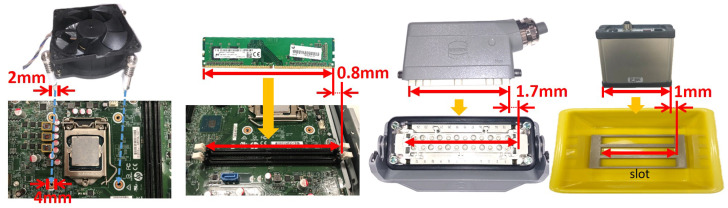
The installation clearances of each task.

To validate the effectiveness of the proposed approach, a series of experiments comprising four tasks: memory card insertion, CPU fan installation, power connector insertion, and a controller packing task, are tested. In the memory card insertion task which inserts the memory card into the slot on the motherboard, in the beginning, the memory card is placed on a stand for the purpose that can be easily grasped by the parallel gripper. The success criteria of each task are defined as follows. The installation tolerances of each task are illustrated in Figure 15. For example, in the CPU fan installation task, the clearance between the pin of the CPU fan and the hole on the motherboard is ±1 mm.

- CPU fan installation: Align four pins of the CPU fan and four holes on the motherboard within ±1 mm.
- Memory card insertion: Insert the memory card into the slot on the motherboard within ±0.8 mm.
- Connector insertion: Insert the connector into the base within ±1.7 mm.
- Controller packing: Place the controller into the slot inside the bin within ±1 mm.

During the demonstration, the human hand movement is recorded at the camera sampling rate of 30 Hz. To segment human motion into action primitives which are Grasp, Move, and Place, the velocity $v_{hand}$ of the hand is used. The timings $t_{grasp}$ for Grasp and $t_{place}$ for Place are determined when $v_{hand}$ < 5 mm/sec. Line feature detection is a crucial step of this approach. Since the visual servoing is only used to fine-tune the final positioning, we retain the line features at the timing $t=t_{place}$ that the object reached the goal position as the target features.

To ensure the robustness and correctness of line segment detection, only the line segments with a length larger than 20 pixels are used for line feature matching. Note that our method does not require any prior knowledge to localize the positions of objects or obstacles. The robot grasps the object and moves it toward the goal position by following the imitated trajectories generated from the human hand movements. The timing to trigger the visual servoing control is determined by the distance to the goal position according to the imitated trajectories. For generalization, the threshold $d_{vf}$ is decided as 1/3 of the distance from the goal position to the highest point of the imitated trajectories in the coordinate system of the table as the visual servoing start timing.

**Figure 16:**
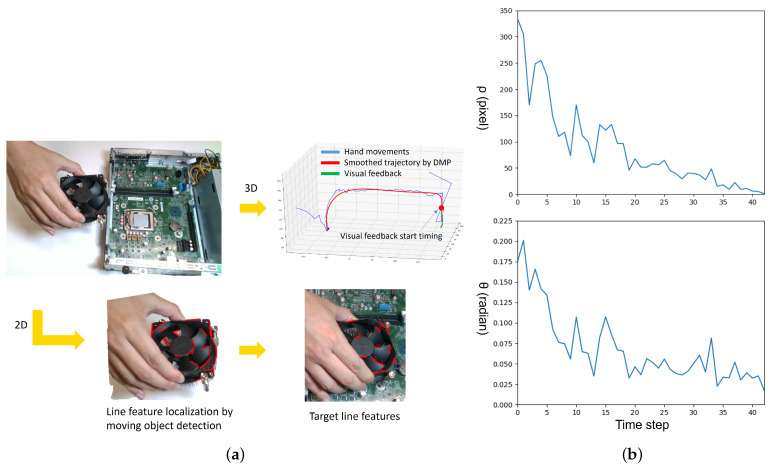

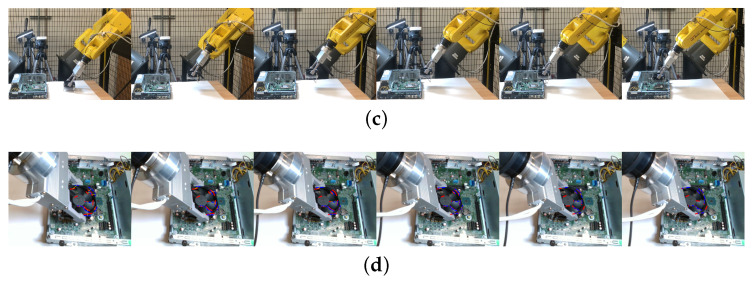
(**a**) CPU fan installation by human demonstration; From human demonstration, 3D imitated trajectories and 2D line features used for visual servoing are generated. (**b**) Line feature error (ρ,θ) between target and current images converge to zero. (**c**) The robot executes a imitated trajectory generated from human demonstration. (**d**) The robot executes a fine-tuned trajectory through the use of visual servoing.

**Figure 17:**
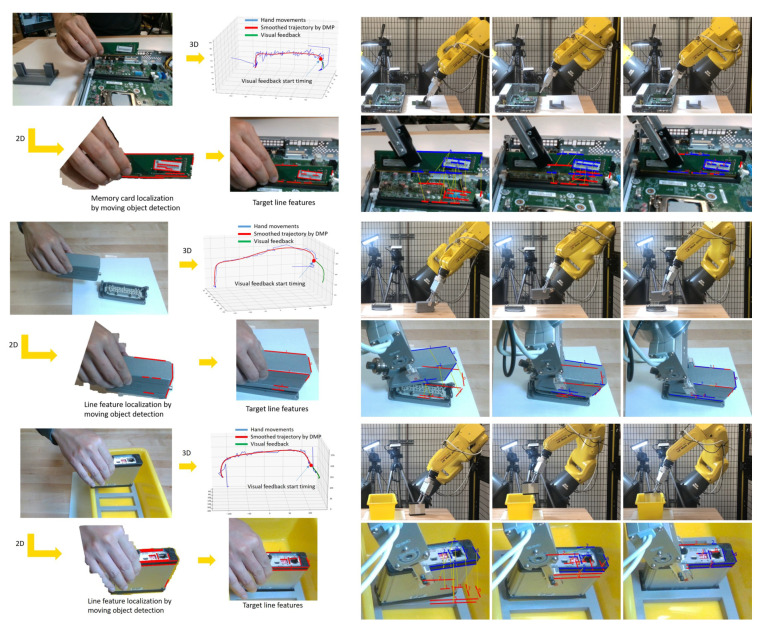
The experiment result. The left side shows the human demonstration phase that 3D imitated trajectories and 2D line features used for visual servoing are generated from human demonstration. The right side shows the robot execution phase which the robot approaches the goal position and switches to visual servoing for precision positioning.

The initial position and the goal position of each task are changed in each trial to better evaluate the performance of the proposed approach. Figure 16 shows the entire process of the CPU fan installation experiment. Figure 17 shows the experiments of the other three tasks. Five trials are performed for each task, and we record the success rates in Table 1. As the baseline, the traditional visual servoing with our proposed line feature matching is used in the comparison. The results show our approach which integrates imitated and fine-tuned trajectories outperforms basic visual servoing. Compared to the visual servoing that often fails with collisions, the imitated trajectories generated from human demonstration significantly increase success rates, especially in complicated environments, such as computer assembly cases. The reinforcement learning-based method may achieve the same high precision as the proposed method. However, reinforcement learning requires a large number of trials that may take four hours to accomplish a connector insertion task [33], and the proposed method achieves a similar task by observing the demonstration only once. We still observed a failure during the experiments. The main reason is due to the collision while executing the visual servoing. Since lots of electronic components exist around the goal position in the computer assembly case, it is easy to collide with the surrounding components during the visual servoing if the displacement of each step is too large. To decrease the parameter α of Equation (16) can obtain a shorter displacement to avoid the collision, but the side effect is that it may increase the cycle time.

## 5. Conclusions

We present a Programming by Demonstration approach that integrates visual servoing techniques to achieve high-precision industrial insertion. Instead of tedious manual feature selection or black-box policy training, our method automatically extracts image features by using the moving object detection function without the need for prior knowledge of the objects. Furthermore, a new line feature matching approach is proposed to overcome the lighting or background-changing problems that are often encountered in traditional texture-based descriptors. Four tasks that include memory card insertion, CPU fan installation, power connector insertion, and a controller packing task are conducted on a FANUC LR Mate 200iD robot to evaluate the performance of the proposed approach, and the results show that high-precision industrial insertion tasks can be executed robustly.

For future works, we plan to utilize different image features rather than the line feature to learn a more general feature-based representative across the different tasks. In addition, this work can be integrated with force control for improving robustness in tight peg-hole-insertion tasks.

## Figures and Tables

**Table 1:** Result of successful trials for each task.

Task	CPU Fan	Memory Card	Connector	Controller
	Success Rate	Success Rate	Success Rate	Success Rate
Visual servoing	1/5	2/5	4/5	2/5
Ours	5/5	4/5	5/5	5/5

## Data Availability

Not applicable.
